# A Diagnostic Challenge: Korsakoff Syndrome Misdiagnosed as Hepatic Encephalopathy

**DOI:** 10.1192/j.eurpsy.2025.1230

**Published:** 2025-08-26

**Authors:** D. Erbil, O. Akay, B. Koparal

**Affiliations:** 1 Psychiatry, Gazi University Faculty of Medicine, Ankara, Türkiye

## Abstract

**Introduction:**

The abrupt occurrence of neuropsychiatric symptoms in patients with alcohol-related cirrhosis requires detailed evaluation not only for hepatic encephalopathy but also for other etiologies like Wernicke-Korsakoff Syndrome. In this case, we present a patient with alcohol-related cirrhosis who was initially admitted to gastroenterology unit with a preliminary diagnosis of hepatic encephalopathy but was ultimately diagnosed with Korsakoff Syndrome.

**Objectives:**

This case highlights the critical need for comprehensive neuropsychiatric assessment in patients with alcohol-related cirrhosis and offers a review of the current literature on the definition and clinical presentation of confabulation.

**Methods:**

Description of a clinical case and literature review

**Results:**

45-year-old male with a history of alcohol-related cirrhosis was admitted to gastroenterology unit with symptoms suggestive of hepatic encephalopathy. On hospital day two, he was consulted to consultation-liaison psychiatry unit with the complaints of sleep disturbances and meaningless talking. In his story; over the past 3 months, he was talking about unrealistic events, having anger outbursts and disrupted sleep cycle, although his total sleep duration remained normal. Aside from grandiosity, mild irritability and disruptions in thought content, there were no signs of manic episode, such as euphoria, distractibility or reduced sleep. In mental state examination, he was alert, oriented in time, place and person, displaying normal psychomotor activity, speech, and impulsivity with euthymic mood. Neurological examination revealed no signs of Wernicke encephalopathy, such as nystagmus, ataxia or confusion. Subsequent psychiatric evaluations revealed fluctuations in narrative, lack of insight, mild memory impairment, and grandiose thought content suggestive of momentary confabulation, all indicative of Korsakoff Syndrome. He was transferred to the psychiatry inpatient unit and treatment with amisulpride at 200 mg daily and thiamin at 300 mg per day were initiated. Following treatment, there was a gradual improvement in aggression; however, no significant enhancement in confabulations or memory function was observed.

**Conclusions:**

To our knowledge, this is the first case report of Korsakoff Syndrome presenting with a preliminary diagnosis of hepatic encephalopathy. The neuropsychiatric components of hepatic encephalopathy can manifest with a variety of symptoms, posing a challenge in differentiation from psychiatric disorders, such as mania or Korsakoff Syndrome. This report highlights the importance of a collaborative evaluation for patients presenting with neuropsychiatric symptoms and a history of alcohol-related cirrhosis.

**Disclosure of Interest:**

None Declared

